# Co-Operative Research

**Published:** 1902-02-15

**Authors:** 


### The Hospital

A Journal of

The Medical Sciences and Hospital Administration.

Vol. XXXI.—No. 803.] Edited by Sir Henry Burdett, K.C.B., and Solomon C. Smith, M.D., M.R.C.P. [February 15, 1902.

### Co-operative Research.

An appeal has lately been made to the public, and has been supported by the names of well-known and highly esteemed members of the medical profession, for a sum of £100,000, to be invested for the purpose of furnishing an income which could be devoted to the conduct of a systematic research into the causes of cancer, in the hope that in this way some light might also be thrown upon the questions of its prevention and its cure. Our non-medical contemporaries have given insertion to many letters in favour of the scheme, and usually containing advice as to the means by which, or the classes from whom, the desired amount might most easily and quickly be obtained. The authorities of numerous hospitals have been eager to point out the wealth, not in money but in "material," of the institutions under their several administrations; and to assert their respective claims to be considered in relation to the matter; while the laboratories of the Conjoint Examining Board on the Embankment have been offered as places in which the work designed may be carried into effect. It is part of the scheme that the actual investigators should be more or less supervised by what are described as "Advisory Committees," and we are apparently asked to believe that the entire project is one which, if only the money were forthcoming, might be carried at once to a successful issue.

We cannot refrain from asking whether such an anticipation is in any degree justified by the history of scientific discovery. It is quite conceivable that an average brewer, or merchant, or banker, or member of the House of Commons, may look upon such discovery as a thing which he has a right to order, as he would order a coat from his tailor, with a corresponding right to complain if it were not delivered with proper punctuality. It seems to be assumed that the way to find out all about cancer is by investigations conducted by the aid of such "material" as can be afforded by patients actually suffering from the disease—a view which, except in so far as it may be possible to gain information by inoculation experiments upon the lower animals, does not commend itself to us as possessing any high degree of inherent probability. It seems far more likely that the initial phenomena of cancer may depend upon some perversion of the series of actions and reactions which those who prefer jargon to knowledge are accustomed to sum up in "the blessed word" metabolism; and, if this be so, a clue to them is not only more likely to be obtained from the apparently healthy than from the manifestly diseased, but is also more likely to be obtained in the course of an inquiry into nutrition in general than in the course of one which has definitely accepted the subject of "cancer" as its goal. We have seen it suggested by the correspondents of non-medical papers that the whole matter is quite simple, and that the discovery and isolation of the "microbe" of cancer ought not to require more than a fortnight or three weeks of the time of an instructed bacteriologist. Whenever the desired knowledge is obtained, it will probably be found that it has been lying unnoticed under the eyes of successive generations of observers; and that the one thing wanted for its utilisation has neither been money, nor a laboratory, nor "material," but simply genius. It has been known for nearly a hundred years, and perhaps for much longer, that malarial fever was not contracted by people who slept under mosquito nets; just as it was known for a hundred years or longer that milkers who had contracted cow-pox were exempt from small-pox. The discovery of vaccination had none the less to wait for Jenner, as the discovery of the action of the anopheles waited for Manson and Ross. Generations had deplored our high rates of inland postage, and had taken endless pains to avoid or evade them, before the attention of Rowland Hill was turned to the subject by an incident which occurred to him during a walk in Cumberland. If the Cancer Fund be established, and the inquirers set to work, it will be in harmony with history if the knowledge for which they seek should ultimately reward the labours of some unaided and previously unappreciated inquirer.

If one part of the projected scheme be more open to criticism than another, it appears to us to be the suggestion of "Advisory Committees." It is not difficult to calculate the precise degree in which the work of Pasteur would have been promoted by an advisory committee of French *savants*, or that of Lord Lister by a similar committee of Edinburgh surgeons. The duty of consultation with a committee would be absolutely fatal to that concentration of individual thought upon a problem to which nearly all great discoveries have been due, and it would be anything but favourable to the strictness of self-examination which all truly scientific work requires. "I suppose," wrote Faraday, "there is scarcely one investigator in original research who has not felt the temptation to disregard the reasons and results which are against his views," and, again, "the world little knows how many of the thoughts and theories which have passed through the mind of a scientific investigator have been crushed in silence and secrecy by his own severe criticism and adverse examination." We do not think that such mental processes, always necessary to the discovery and establishment of truth, would be promoted by the co-operation of a committee. Its only effect would be to compel the worker to defend his immature conclusions against all and sundry, and to seek victory over his advisory colleagues, instead of seeking only to defend himself against error, and to hope for no victory but that of truth.

